# Prevalence and seroprevalence of *Plasmodium* infection in Myanmar reveals highly heterogeneous transmission and a large hidden reservoir of infection

**DOI:** 10.1371/journal.pone.0252957

**Published:** 2021-06-09

**Authors:** Hannah M. Edwards, Ruth Dixon, Celine Zegers de Beyl, Olivier Celhay, Mousumi Rahman, Moe Myint Oo, Thandar Lwin, Zaw Lin, Thiri San, Kay Thwe Han, Myaing Myaing Nyunt, Christopher Plowe, Gillian Stresman, Tom Hall, Chris Drakeley, Prudence Hamade, Siddhi Aryal, Arantxa Roca-Feltrer, Thaung Hlaing, Aung Thi

**Affiliations:** 1 Malaria Consortium, Bangkok, Thailand; 2 Malaria Consortium, Yangon, Myanmar; 3 Malaria Consortium, London, United Kingdom; 4 Ministry of Health and Sports, Yangon, Myanmar; 5 Parasitology Research Division, Department of Medical Research, Yangon, Myanmar; 6 University of Maryland School of Medicine, Baltimore, Maryland, United States of America; 7 Faculty of Infectious and Tropical Diseases, London School of Hygiene and Tropical Medicine, London, United Kingdom; Ehime Daigaku, JAPAN

## Abstract

Malaria incidence in Myanmar has significantly reduced over recent years, however, completeness and timeliness of incidence data remain a challenge. The first ever nationwide malaria infection and seroprevalence survey was conducted in Myanmar in 2015 to better understand malaria epidemiology and highlight gaps in Annual Parasite Index (API) data. The survey was a cross-sectional two-stage stratified cluster-randomised household survey conducted from July-October 2015. Blood samples were collected from household members for ultra-sensitive PCR and serology testing for *P*. *falciparum* and *P*. *vivax*. Data was gathered on demography and a priori risk factors of participants. Data was analysed nationally and within each of four domains defined by API data. Prevalence and seroprevalence of malaria were 0.74% and 16.01% nationwide, respectively. Prevalent infection was primarily asymptomatic *P*. *vivax*, while *P*. *falciparum* was predominant in serology. There was large heterogeneity between villages and by domain. At the township level, API showed moderate correlation with *P*. *falciparum* seroprevalence. Risk factors for infection included socioeconomic status, domain, and household ownership of nets. Three K13 *P*. *falciparum* mutants were found in highly prevalent villages. There results highlight high heterogeneity of both *P*. *falciparum* and *P*. *vivax* transmission between villages, accentuated by a large hidden reservoir of asymptomatic *P*. *vivax* infection not captured by incidence data, and representing challenges for malaria elimination. Village-level surveillance and stratification to guide interventions to suit local context and targeting of transmission foci with evidence of drug resistance would aid elimination efforts.

## Introduction

The incidence of malaria in Myanmar has reduced significantly in recent years, falling by over 80% from a reported 1341.8 cases per 100,000 population in 2005 to 253.3 cases per 100,000 population in 2014 [1]. Similarly, malaria mortality fell by over 90% from 3.79 deaths per 100,000 to 0.25 per 100,000 over the same period. This trend reflects a corresponding increase in political and financial commitment from the Myanmar government and partners. Efforts resulted in strengthened malaria prevention and case management interventions, including deployment of village health workers (VHWs) and large-scale long-lasting insecticide-treated net (LLIN) distribution. Along with the rest of the Greater Mekong Sub-region (GMS) countries, the Myanmar national malaria control programme (NMCP) has drafted a strategy to eliminate malaria by 2030 [2, 3]. Despite this, Myanmar has the highest incidence of any country in the GMS, evidence of artemisinin resistance, challenges of highly mobile population groups, remote and hard-to-reach areas of high transmission and outdoor biting vectors presenting obstacles to meeting this malaria elimination goal [4–7]. Completeness and timeliness of collation of surveillance data remains a significant challenge in some regions, particularly those that are remote and the true incidence of malaria in the country may, therefore, be underestimated [8].

Prevalence surveys can highlight gaps in epidemiological understanding and if conducted regularly can measure the rate of impact of interventions on decreasing transmission. There has been increasing interest in the use of highly sensitive infection tests and serological methods in monitoring malaria transmission intensity in low transmission settings where prevalence is low since a large proportion of infections are asymptomatic and low density and thus missed by conventional diagnostic methods [9, 10]. These methods can also provide detail on within country heterogeneity of infection when analysed alongside spatial data and have the potential to inform control and elimination programmes.

Here, we present results from this survey in relation to geography, incidence data and associated risk factors to better understand the epidemiology of malaria in Myanmar and the challenges faced by drug resistance and ultimately malaria elimination.

## Methods

The MIS protocol, questionnaire and tools were submitted to the Ethics Review Committee on Medical Research Involving Human Subjects, Department of Medical Research. Following defence of the submission full ethical approval was granted by the committee on 21/07/2015 (Letter Number: 59/ Ethics 2015, dated 21/07/2015)."

### Study area

Myanmar (Burma) sits within the GMS, sharing borders with Thailand, China, India and Bangladesh. In 2015, Myanmar had an estimated population of 53,900,000, and an estimated malaria at-risk population of 32,120,000 residing in 15 malaria endemic states/regions [11].

### Study design and sampling procedure

**Fig 1:**
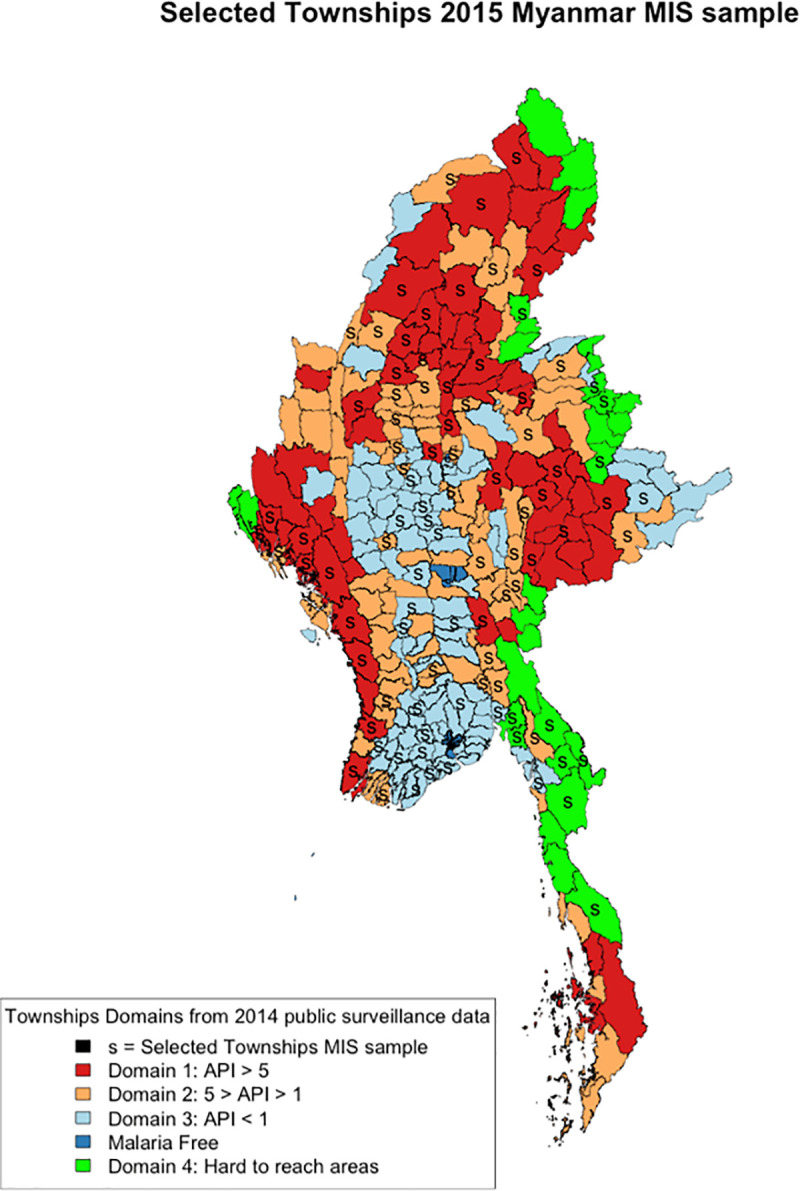
Map of Myanmar showing location of the four sampling domains. Domain 1: API>5; Domain 2: API 1–5; Domain 3: API<1; Domain 4: non-state areas.

The Myanmar Malaria Indicator Survey (MIS) was a national cross-sectional household survey conducted from July to October 2015. The survey used a two-stage stratified cluster-randomised design, with a cluster defined as a ‘village’ and a ‘household’ as the sample unit. All malaria endemic townships were included and were categorised into four non-contiguous strata, termed domains, three based on intensity of malaria transmission using API data from the township level (Domain 1—API >5; Domain 2—API 1–5; Domain 3—API <1) and non-state areas included in a separate fourth domain due to limited incidence data and variable administrative structures (Fig 1).

Townships were randomly selected with probability proportionate to size (PPS) and with replacement to ensure the required number of clusters within each domain was reached. One village tract/ward (VT/W) per selected township was randomly selected with uniform probability. In each selected VT/W, field teams updated village level population and one village per VT/W was randomly selected by PPS. In each selected village, field teams updated household listings to include all registered and non-registered households at the time of the survey and then randomly selected 31 households for participation. In areas covered by Domain 4 (non-state areas), administrative systems were independent and unique. In each area, the survey team interposed the government of Myanmar township boundaries and made random selection of townships with PPS. Available population data supplied by non-state actors was then used to make random selection with PPS of villages per township. Households were selected in the same manner as in the other domains. Certain areas that were absolutely inaccessible at the time of surveying due to active conflict or flooding were excluded from the sampling frame.

### Sample size

Sample size was calculated based on assumed prevalence levels detectable by PCR in each of the strata and using the standard formula for proportions, assuming a minimum precision of 0.01, a non-response rate of 10%, a design effect of 2.0, average household size of 4.5 and selection of 31 households per cluster. Based on this, a total 20,138 individuals from 4,495 households and 145 clusters across the four domains were required.

### Data collection

Prior to data collection, fully informed consent was gathered from the head of the household on behalf of all household members and for children, where applicable, for both questionnaire information and blood collection. Study information was provided in written and/or verbal form in a language understandable to the participant and written consent was provided by signature or thumb print.

In each selected household, a standard questionnaire was administered to the head of the household or his/her representative. The questionnaire was designed to gather data on factors related to demographic background, including age, sex and wealth of the household; malaria risk factors, including fever, travel and forest-going habits; net ownership and usage, including type of net; case management practices, including healthcare seeking behaviour; and knowledge of malaria. These data were gathered on all household members, including temporary visitors staying in the household at the time of the survey. A traveller was defined as a household member who has travelled and stayed overnight away from home in the previous six months. A forest-goer was defined as a household member who sometimes goes to the forest and stays there overnight.

Upon obtaining informed consent, all household members aged over six months present in the households were tested for malaria infection by rapid diagnostic test (RDT) (brand: SD Bioline Pan *P*. *falciparum*/*P*. *vivax*) and asked to provide four dried blood spots on filter paper for subsequent PCR, K13 molecular analysis and serological testing. Blood spots from each individual from a finger prick using a sterile lancet and necessary measures to ensure sterile blood collection (alcohol wipes, cotton wool, etc.). The survey team revisited households up to three times to get blood samples from all household members. GPS coordinates were recorded in every sampled household and from a central point in each cluster to assess for geographical clustering of infection, except in domain 4 where no geolocation data were provided due to local sensitivities.

### Laboratory analyses

Two filter paper samples (one FTA Elute card containing two bloodspots for PCR and K13; and one 3MM Whatman card containing one bloodspot for serology) and one extra blood spot were prepared for each participant. Samples were labelled using the CDC bar coding system. All blood samples were collected using individual capillary tubes, spotting the samples on Whatman 3MM filter paper as described [12]. DBS samples were air-dried, placed in a sealed plastic pouch with desiccant and stored at room temperature until analysed in the laboratory (up to 6 months).The DBS were used for: 1) detection of malaria species and, 2) seroprevalence analysis. Nucleic acids were extracted from dried blood spots using an optimized protocol as described by Zainabadi et al. [12]. The prevalences of the two major *Plasmodium* species (*P*. *falciparum* and *P*. *vivax*) were estimated using ultrasensitive PCR with a detection limit of 20 parasites per millilitre, as previously described by Adams et al. [13] *P*. *falciparum* positive samples were subsequently tested for mutations in the Kelch gene on chromosome 13 (K13), a molecular marker for artemisinin resistance, as described in Ouattara *et al*. [14].

For serological analysis, dry blood spots were eluted and tested for IgG antibodies to *P*. *falciparum* and *P*. *vivax* AMA1 and MSP1-19 as previously described [15]. Briefly, sera were eluted from 3mm dried blood spots and prepared as a 1/200 dilution in 1XPBS/0.05% tween. Each antigen was coated to microtitre plates (Immulon4) and incubated overnight at 4°C. Following a wash step (3 times in PBS-T) the plates were blocked for 3 hours with 1xPBS/0.05%Tween/1% milk powder. Following a further wash step samples were added in duplicate at a dilution of 1/1000 (MSP) and 1/2000 (AMA). Species specific positive sera were used on each plat and to enable the adjustment of results to account for plate to plate variation; for *P*.*falciparum*, CP3 a hyper immune positive pool from Tanzania and for P.vivax, 72/096 a standard with high reactivity to *P*.*vivax* obtained from NIBSC. Plates were then incubated overnight at 4°C. The following day the plates were washed 5 times and incubated for 3 hours with an anti-human IgG-HRP (DAKO) diluted to 1/5000, for 3 hours. The plates were washed a further 5 times and incubated for 20 minutes with Sigmafast OPD for 20 minutes in the dark, the plates were then read at 492 nm. A titration curve was fitted to the ODs obtained for the standard plasma dilutions by least squares minimisation using a three variable sigmoid model and the solver add-in in Excel (Microsoft), assuming an arbitrary value of 1000 Units/ml of antibody against each antigen in the standard pool. OD values were converted to units/ml using this fitted curve [15]. Cut-offs to define antigen specific seropositivity were generated using the mixture model and analysed using standard measures [16, 17]. A sample was considered positive if OD values were above the seropositivity threshold for at least one of the two antigens.

### Data analysis

Data from paper-based questionnaires were double entered into EpiData and analysed using Stata 14.1 with figures produced in R 3.4.2 [18, 19]. Analysis was adjusted using the appropriate weights for households and individuals, respectively according to the study design. Descriptive statistics were gathered on demographics of the sample population and of malaria infection and exposure, disaggregated by species, calculated nationally and per domain. Socioeconomic status of HHs were assessed by splitting HHs into wealth quintiles, with quintile 1 (Q1) as the poorest 20% of HHs, and quintile 5 (Q5) as the wealthiest 20% of HHs. All proportions were calculated with 95% confidence intervals (CI) to indicate significant differences in estimates both between and within domains.

Since RDT positivity rates were low, primary outcomes were PCR prevalence and seroprevalence of *P*. *falciparum* and *P*. *vivax*. Using available GPS coordinates, clusters were mapped according to prevalence and seroprevalence of *P*. *falciparum* and *P*. *vivax* to explore potential geographic clustering of infection. To maintain anonymity in Domain 4, clusters were mapped to the GPS coordinates of the central township to which they belonged as available from Google Maps and Wikipedia [20, 21].

To compare routine malaria incidence with survey prevalence data, cluster prevalence data was aggregated at the township level and prevalence/seroprevalence was plotted against township API (defined as the number of malaria cases per 1000) data collected during 3-month design phase of the survey (e.g. Domain classification of townships). For each plot a Loess curve was generated to characterise the relationship between prevalence and API.

Risk factor analysis was conducted using logistic regression modelling with PCR positive/seropositive *P*. *falciparum* or *P*. *vivax* infection as the dependent variable. Crude odds ratios (ORs) and 95% CI for each species of infection were generated from independent variables related to demographic factors, net coverage and usage, history of fever, healthcare-seeking behaviours, risk group categories and knowledge indicators of the head of the household. Variables with a p-value ≤0.05 were included in a multivariable model and step-wise backwards deletion of variables was done until a final multivariable model was generated with only significant associations included. Age and sex were controlled for in all final models.

## Results

A total of 4,371 households comprising 20,638 household members were included in the survey (Table 1). Blood samples were collected from 13,873 (67%) household members for PCR analysis of which 13,726 could be matched to cluster data and 13,716 to individual level data. The 157 (1.13%) samples unable to be matched were due to a loss of questionnaire data from one cluster and incorrect identifiers on some blood samples. The four DBS were prioritised for PCR and K13 testing, so if a DBS did not have enough material for testing, the next spot would be used for PCR/K13, while remaining DBS were used for serological analysis. This resulted in 11,653 blood samples available for serological analysis.

**Table 1:** Sample size achieved across domains.

	Domain 1	Domain 2	Domain 3	Domain 4	Total
No. of households	1,734	986	757	894	4,371
No. of people in households	8,545	4,544	3,251	4,298	20,638
No. (%) of household members with PCR sample*	5,687 (66.6)	3,121 (68.7)	2,254 (69.3)	2,664 (62.0)	13,726 (66.5)
No. (%) of household members with serology sample	4,685 (54.8)	2,724 (60.0)	2020 (62.1)	2,224 (51.8)	11,653 (56.5)

*10 samples could be matched to cluster but not to individual questionnaire data, so for individual analysis n = 13,716

Among all household members, there was no difference in the proportion that gave blood samples by domain, wealth quintile or urban/rural area, though there was variation in the level of participation of individual clusters (ranging from 18.9% to 93.0% per cluster) and several clusters achieved less than 50% of household members contributing blood samples to the analysis, particularly in Domain 4 (S1 Table). Significantly fewer males, children aged less than five years and travellers contributed blood samples for PCR analysis. For serology, significantly fewer travellers, individuals with any schooling, and children aged less than 5 years provided blood samples for analysis. Pregnant women were the most covered group for both diagnostic procedures (S1 Table).

### Prevalence and seroprevalence

**Fig 2:**
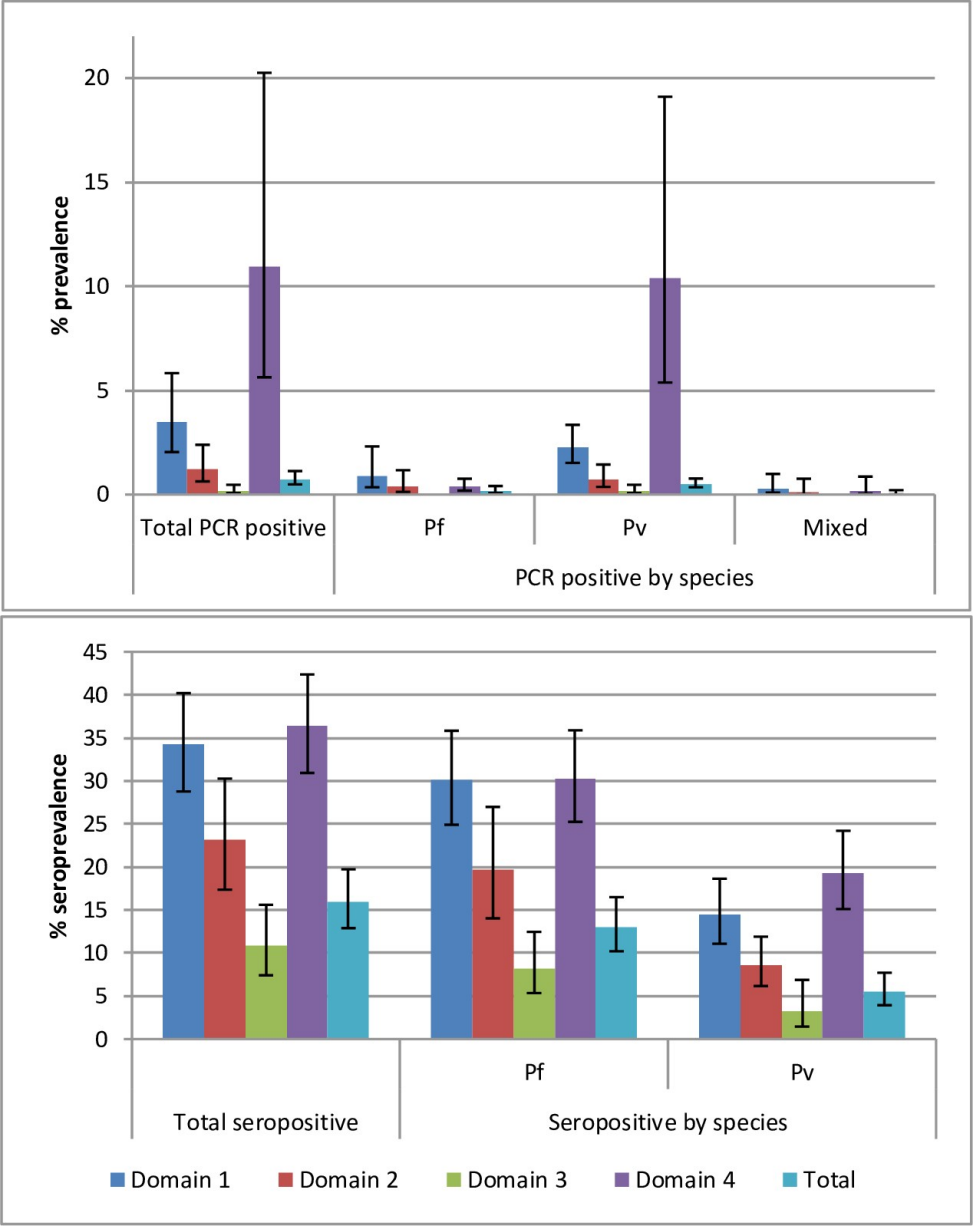
**usPCR prevalence (top) and seroprevalence (bottom) of *P*. *falciparum*, *P*. *vivax* and mixed infection in Myanmar nationwide and within each of four domains.** Mixed infection not included in separate *P*. *falciparum/P*. *vivax* prevalence figures.

Of 13,648 RDTs conducted, only 10 positive cases were identified including eight *P*. *falciparum* and two *P*. *vivax* infections. Since the number of cases was so low, this was not included as a primary outcome in subsequent analyses. Overall, PCR prevalence of *Plasmodium* infection was 0.74%, 95% CI [0.49–1.13], but was significantly higher in Domain 4 at 10.96%, 95% CI [5.63–20.25], followed consecutively by Domain 1, Domain 2 and finally Domain 3 (Fig 2). *P*. *vivax* made up 70% of infections. Prevalence of *P*. *vivax* was 0.52%, 95% CI [0.35–0.77] overall, but significantly higher in Domain 4 (10.39%, 95% CI [5.38–19.10]) followed by Domain 1 (2.26%, 95% CI [1.52–3.35]). Prevalence of *P*. *falciparum* was low at only 0.17%, 95% CI [0.07–0.41] and did not differ significantly by domain. No *P*. *falciparum* cases were detected in Domain 3. There were 28 mixed infections identified, giving an overall prevalence of 0.05%, 95% CI 0.01–0.21, but the majority (n = 20) were detected in Domain 1.

**Fig 3:**
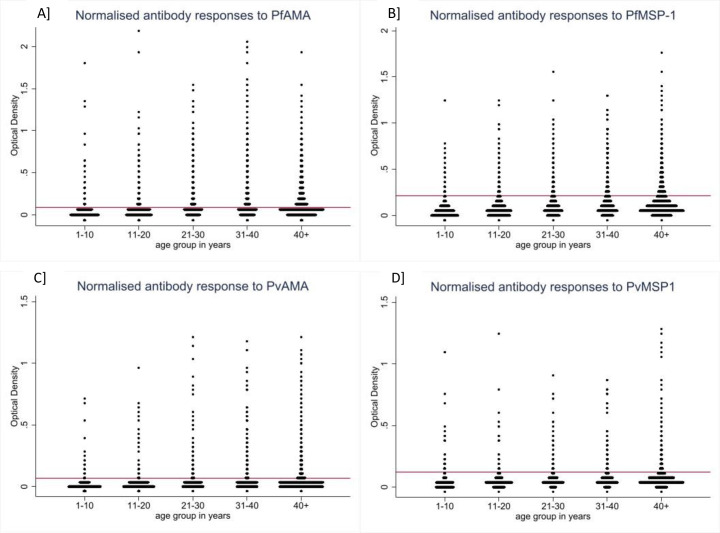
Normalised age-stratified antibody responses to *P*. *falciparum* antigens A] PfAMA, B] PfMSP-1, and *P*. *vivax* antigens C] PvAMA, D] PvMSP-1. Horizontal red lines represent antigen-specific cut-off valus.

Seroprevalence was 16.01%, 95% CI [12.89–19.72] overall and was highest in Domains 4 and 1 (36.45%, 95% CI [30.92–42.36] and 34.26%, 95% CI [28.77–40.19], respectively) and significantly lowest in Domain 3 (10.84%, 95% CI [7.41–15.60], Fig 2). *P*. *falciparum* seroprevalence was over two-times higher than *P*. *vivax*. *P*. *falciparum* seroprevalence was significantly lower in Domain 3 and appeared highest in Domains 1 and 4 though CIs overlapped with Domain 2. *P*. *vivax* seroprevalence was highest in Domain 4, followed consecutively by Domain 1, 2 and finally 3 (though CIs overlap between each level). Antibody responses split by age showed there were individuals with marked antibody responses in all age groups, including children aged 1–10 (Fig 3).

### K13

Of the total 80 *P*. *falciparum* infections identified, 17 had high enough parasite load to qualify for K13 testing. Of these, three (17.7%) had a mutation in the F446I gene, each from three different clusters in Domain 1, two of which were in Pinlebu township, north-western Myanmar.

### Mapping of clusters

**Fig 4:**
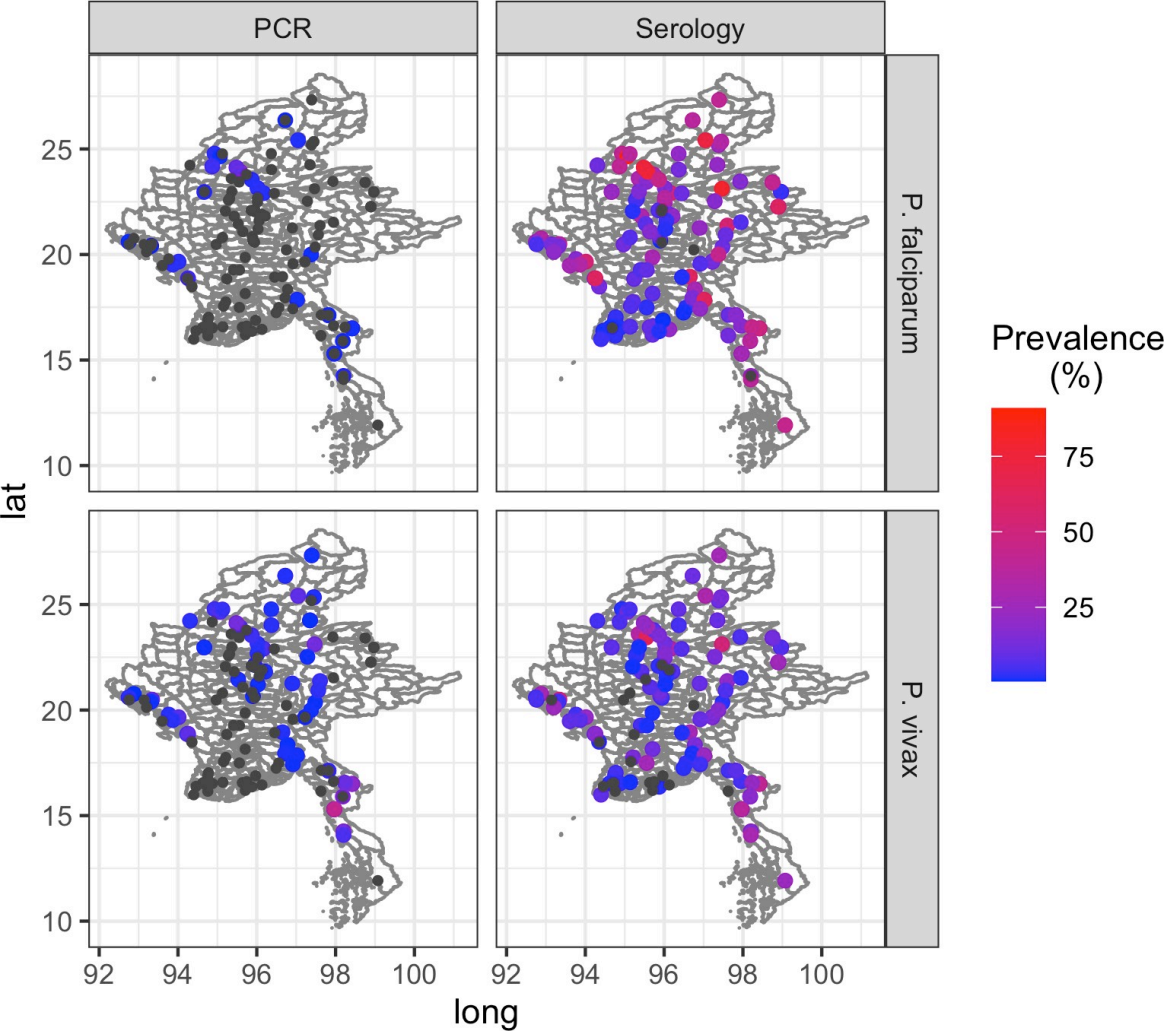
**Maps showing prevalence (left column) and seroprevalence (right column) of *P*. *falciparum* (top row) and *P*. *vivax* (bottom row) in each sampled cluster.** Each dot represents one cluster (one village) coloured according to level of prevalence or seroprevalence. Black dots indicate clusters where no positive PCR or serology cases were identified.

Clusters (villages) were plotted based on prevalence and seroprevalence of both *P*. *falciparum* and *P*. *vivax* (Fig 4). Clusters of higher *P*. *falciparum* prevalence were observed in Sagaing Region, north-west Myanmar (≈latitude 24^o^ longitude 95.5^o^). Two of these clusters were from the same township, Pinlebu, with prevalences of 18.33% and 9.49%, one of which also contained a K13 mutant. These were followed by lower prevalences in another township of Sagaing (4.05%) and two clusters in Ann township of Rakhine State, south-west Myanmar (2.33% and 2.20%, ≈latitude 19^o^ longitude 94^o^). The highest prevalence clusters for *P*. *vivax* were in Domain 4, including 60.34% from a cluster in Yephyu township, Thanintharyi State; 42.40% and 29.91% from two clusters in Yay township, Mon State; and 19.79% from a cluster in Myawaddy township, Kayin State (all in the south-east tip of Myanmar).

Seroprevalence was higher and more widespread throughout the country particularly for *P*. *falciparum*. Although there were many clusters without any evidence of infections by PCR (coloured in black), there were only few clusters without any seropositive individuals.

### Asymptomatic infection and risk factors for infection

The vast majority of PCR-positive *P*. *vivax* infections were asymptomatic, defined as not having current fever nor reported fever in the previous two weeks (99.53%, 95% CI [94.84–99.81]) and in Domains 2 and 3, all *P*. *vivax* infections were asymptomatic. *P*. *falciparum* had a higher proportion of symptomatic infection compared to *P*. *vivax* (22.99%, 95% CI [6.49–56.22] versus 0.47%, 95% CI [0.19–1.12], Pearson’s chi-square = 82.3, p<0.001).

**Fig 5:**
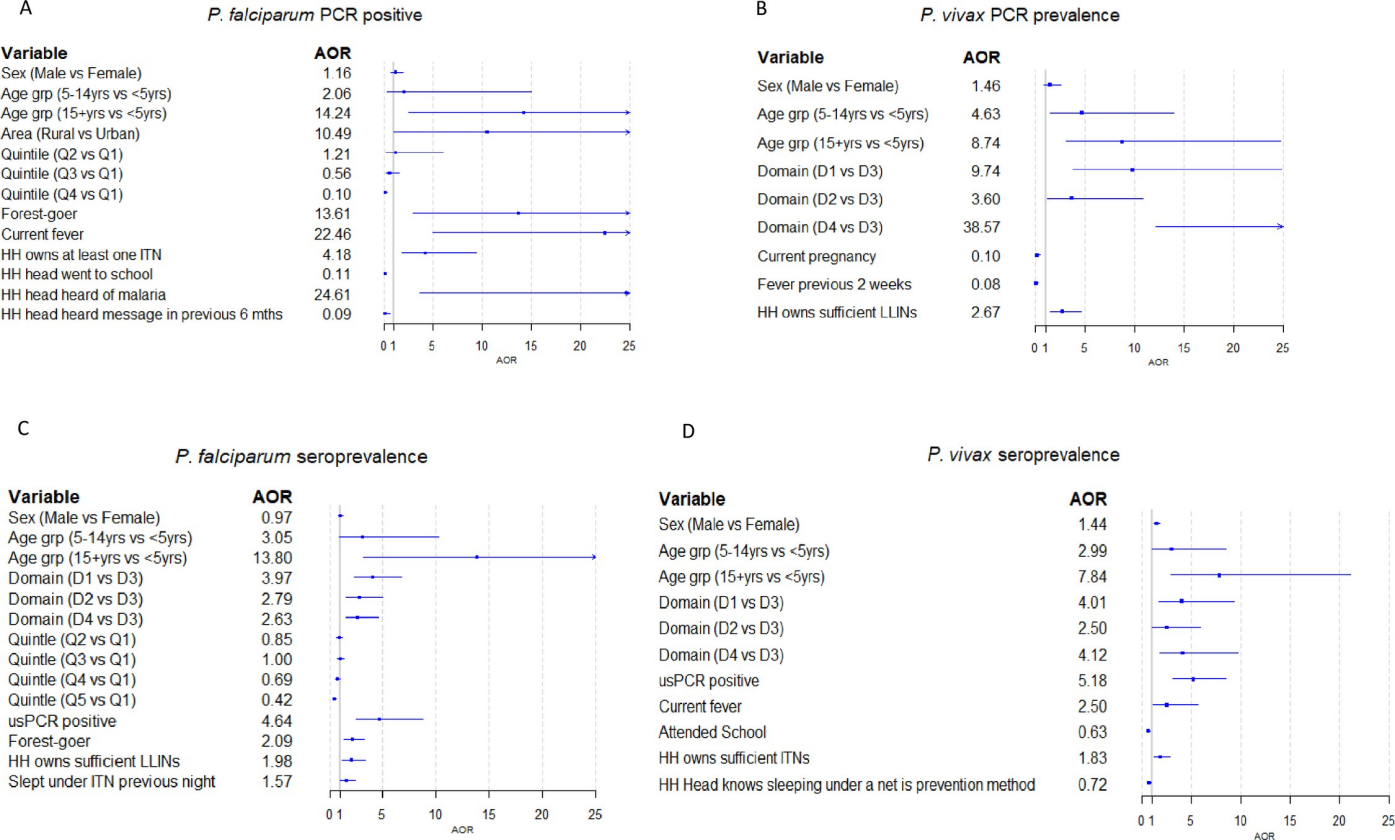
Forest plots showing Adjusted Odds Ratios (AOR) for risk factors related to being positive by usPCR or by serology across the whole study sample (n = 13,716 for PCR and 11,653 for serology). A] Prevalence of *P*. *falciparum*, B] PCR prevalence of *P*. *vivax*, C] Seroprevalence of *P*. *falciparum*, D] Seroprevalence of *P*. *vivax*. Factors shown are those that remained significant in multivariate logistic regression analyses (plus sex and age group).

Risk factors for PCR positivity and seropositivity among the whole population sample differed slightly between *Plasmodium* species and per domain (Fig 5). PCR prevalence of *P*. *falciparum* was associated with older age groups, wealth quintile, staying in the forest and with current fever. It was also associated with HH ownership of bed nets and with knowledge of malaria. *P*. *vivax* prevalence was associated with age, domain, absence of fever and owning sufficient nets, and was lower in pregnant women.

As expected, seropositivity increased with age, and whilst there were still seropositive children aged less than 5 years for both *P*. *falciparum* (prevalence in under-fives ranging from 1.2 to 3.1% across the 4 domains) and *P*. *vivax* (ranging from 2.2 to 3.7% across domains) the majority of these had antibody levels close to the seropositivity threshold. Seroprevalence of *P*. *falciparum* was also associated with wealth quintile, staying in the forest and ownership of nets, as well as domain, and being PCR positive (Fig 5). *P*. *vivax* seroprevalence was also associated with age and domain, as well as being PCR positive, having current fever, ownership of nets and knowledge of nets (Fig 5).

Analysis of the whole population sample, however, hides interesting differences in risk factors between domains for both prevalence and seroprevalence of each species (Tables 2–5). For example, *P*. *falciparum* infection was associated with current pregnancy in Domain 1, with current fever and staying in the forest in Domain 2, and with being a traveller in Domain 4 (Table 2). *P*. *vivax* infection was associated with being a forest-goer in Domain 1 and 4, but not in Domain 2 (Table 3). In Domain 4, being from a rural area or lower wealth quintile was also associated with P. vivax infection but these were not significant in the other domains. For *P*. *falciparum* seropositivity, wealth quintile was a significant factor across all domains, but the importance of sex and age differed with males having higher odds of being seropositive in domains 1 and 4, but not domains 2 or 3, and age being significant in domains 1, 2, and 3 but not domain 4 (Table 3). *P*. *falciparum* seropositivity was also associated with living in a rural area in domain 4, being a forest-goer in domain 3, and having current fever in domain 1. Similarly for *P*. *vivax* seropositivity, the importance of sex differed between domains, as well as wealth quintile (domain 1), current fever (domain 3) and living in a rural area (domain 4). Interestingly, higher odds of infection and exposure indices were associated with higher ITN/LLIN ownership and/or knowledge of ITN as a prevention tool for both plasmodia species and in most domains.

**Table 2:** Domain specific adjusted ORs for *P*. *falciparum* infection^1^.

				Crude OR			Adjusted OR		
			Prevalence, % (95% CI)	OR	95% CI	p-value	OR	95% CI	p-value
**D1**	**Sex**	**Female**	0.80 (0.27–2.37)	1			1		
		**Male**	1.04 (0.44–2.46)	1.31	0.82–2.11	0.3	1.44	0.88–2.36	0.1
	**Age group**	**<5**	0.27 (0.04–2.00)	1			1		
		**5 to 14**	0.75 (17–3.28)	2.80	0.42–18.60	0.3	2.95	0.45–19.48	0.3
		**15+**	1.00 (0.40–2.46)	3.7	1.03–13.31	0.05	3.59	1.00–12.87	0.05
	**Current pregnancy**		2.31 (0.60–8.41)	2.66	1.40–5.06	0.004	3.09	1.61–5.95	0.001
	**HH owns at least one ITN**		1.31 (0.41–4.05)	5.18	1.23–21.80	0.03	4.75	1.13–20.01	0.03
	**HH head went to school **		0.58 (0.25–1.32)	0.33	0.18–0.61	0.001	0.36	0.19–0.66	0.001
**D2**	**Sex**	**Female**	0.33 (0.12–0.87)	1			1		
		**Male**	0.48 (0.12–1.88)	1.49	0.60–3.65	0.4	1.06	0.52–2.15	0.9
	**Age (per year)**		n/a	1.13	1.04–1.23	0.005	1.07	0.94–1.21	0.3
	**Current fever**		5.71 (1.91–15.86)	20.85	2.98–145.70	0.003	35.42	6.03–207.86	< .001
	**Forest-goer**		5.81 (1.08–25.95)	33.05	5.02–217.48	0.001	42.15	4.37–406.20	0.002
	**Head of HH went to school **		0.10 (0.02–0.40)	0.07	0.03–0.19	< .001	0.08	0.03–0.22	< .001
**D4**	**Sex**	**Female**	0.17 (0.06–0.51)	1			1		
		**Male**	0.64 (0.27–1.52)	3.72	1.00–13.88	0.05	3.13	0.92–10.59	0.07
	**Age group**	**<5**	0.30 (0.04–2.52)	1			1		
		**5 to 14**	0.40 (0.12–1.31)	1.32	0.11–15.41	0.8	1.34	0.12–15.17	0.8
		**15+**	0.39 (0.17–0.86)	1.27	0.12–12.93	0.8	1.00	0.09–10.55	1
	**Traveller**		3.00 (0.77–11.00)	10.47	2.39–45.88	0.003	8.21	1.90–35.36	0.006
	**HH head knows ITN is prevention method**		1.57 (0.35–6.73)	5.01	1.03–24.37	0.05	4.38	1.07–17.92	0.04

^1^Models for domain 3 were not able to be completed due to lack of sufficient infections

**Table 3:** Domain specific adjusted ORs for *P*. *vivax* infection^1^.

			Prevalence % (95%CI)	Crude OR			Adjusted OR		
				OR	95% CI	p-value	OR	95% CI	p-value
**D1**	**Sex**	**Female**	1.25 (0.78–2.00)	1			1		
		**Male**	3.65 (2.44–5.43)	2.98	2.19–4.06	< .001	3.04	2.18–4.24	< .001
	**Age group**	**<5**	0.81 (0.18–3.55)	1			1		
		**5 to 14**	1.54 (0.68–3.43)	1.90	0.33–11.10	0.5	2.02	0.35–11.61	0.4
		**15+**	2.59 (1.79–3.75)	3.24	0.70–15.03	0.1	3.41	0.76–15.34	0.1
	**Forest-goer**		7.83 (3.50–16.57)	4.01	1.78–9.01	0.001	2.42	1.07–5.46	0.04
	**HH owns sufficient LLINs**		5.02 (2.98–8.34)	3.95	2.14–7.31	< .001	3.90	2.12–7.16	< .001
	**HH head knows sleeping under net is prevention method**		1.68 (1.06–2.63)	0.58	0.38–0.87	0.01	0.58	0.39–0.88	0.01
**D2**	**Sex**	**Female**	0.53 (0.24–1.19)	1			1		
		**Male**	0.99 (0.49–1.99)	1.87	1.09–3.22	0.02	1.96	1.13–3.40	0.02
	**Age (per year)**		n/a	1.01	0.98–1.03	0.6	1.09	0.98–1.20	0.1
	**HH owns sufficient LLINs**		1.65 (0.66–4.06)	2.86	1.11–7.35	0.03	2.97	1.13–7.83	0.03
**D4**	**Sex**	**Female**	10.56 (4.50–22.82)	1			1		
		**Male**	10.06 (6.09–16.17)	0.95	0.57–1.57	0.8	0.89	0.51–1.56	0.7
	**Age group**	**<5**	4.94 (1.27–17.30)	1			1		
		**5 to 14**	12.48 (4.73–29.07)	2.74	1.58–4.76	0.001	1.75	0.81–3.77	0.1
		**15+**	10.09 (5.86–16.82)	2.16	0.83–5.60	0.1	1.28	0.41–3.93	0.7
	**Area**	**Rural**	12.13 (6.03–22.90)	10.87	2.46–48.05	0.003	9.23	1.99–42.78	0.006
	**Quintile**	**Q1 (poorest)**	12.28 (5.69–24.51)	1			1		
		**Q2**	12.04 (6.72–20.62)	0.98	0.63–1.52	0.9	0.85	0.55–1.33	0.5
		**Q3**	8.06 (3.66–16.81)	0.63	0.35–1.12	0.1	0.62	0.36–1.06	0.08
		**Q4**	8.44 (3.63–18.42)	0.66	0.35–1.25	0.2	0.70	0.37–1.34	0.3
		**Q5**	0.93 (0.10–7.94)	0.07	0.01–0.58	0.02	0.06	0.01–0.51	0.01
	**Forest-goer**		18.06 (10.13–30.12)	2.02	1.22–3.35	0.008	2.10	1.37–3.20	0.001
	**Attended school**		12.64 (6.25–23.91)	2.00	1.31–3.05	0.002	1.90	1.20–3.01	0.008
	**HH owns sufficient ITNs**		13.59 (7.33–23.83)	2.12	1.19–3.79	0.01	1.69	1.02–2.80	0.04

^1^Models for domain 3 were not able to be completed due to lack of sufficient infections

**Table 4:** Crude and adjusted odds ratios for *P*. *falciparum* seropositivity per domain.

				Crude OR			Adjusted OR		
			Prevalence % (95% CI)	OR	95% CI	p-value	OR	95% CI	p-value
**D1**	**Sex**	**Female**	27.57 (22.12–33.77)	1			1		
		**Male**	33.43 (28.26–39.03)	1.32	1.15–1.51	< .001	1.47	1.25–1.72	< .001
	**Age group**	**<5**	3.01 (0.73–11.57)	1			1		
		**5 to 14**	10.48 (6.83–15.75)	3.77	0.80–17.79	0.09	5.06	1.25–20.47	0.02
		**15+**	36.61 (29.68–44.15)	18.6	4.44–77.93	< .001	29.66	8.04–109.41	< .001
	**Wealth quintile**	**Q1**	31.92 (26.28–38.13)	1			1		
		**Q2**	35.47 (27.73–44.04)	1.17	0.88–1.57	0.3	1.05	0.73–1.53	0.8
		**Q3**	30.59 (22.46–40.12)	0.94	0.68–1.29	0.7	0.79	0.53–1.16	0.2
		**Q4**	27.94 (21.01–36.12)	0.83	0.57–1.21	0.3	0.66	0.42–1.05	0.08
		**Q5**	23.73 (19.51–28.55)	0.66	0.48–0.92	0.02	0.57	0.37–0.88	0.01
	**PCR positive**		71.54 (58.95–81.49)	6.23	3.73–10.41	< .001	4.76	2.61–8.69	< .001
	**Current Fever**		46.13 (33.37–59.42)	2.02	1.21–3.36	0.008	2.43	1.49–3.98	0.001
	**HH owns sufficient ITNs/LLINs**		39.39 (30.44–49.10)	1.78	1.23–2.57	0.003	1.72	1.14–2.61	0.01
**D2**	**Sex**	**Female**	19.21 (13.23–27.06)	1			1		
		**Male**	20.50 (14.58–28.05)	1.08	0.90–1.30	0.4	1.17	0.96–1.42	0.1
	**Age group**	**<5**	3.08 (1.00–9.10)	1			1		
		**5 to 14**	5.48 (2.97–9.88)	1.83	0.71–4.73	0.2	3.6	0.74–17.47	0.1
		**15+**	23.90 (16.39–33.48)	9.89	3.05–32.11	< .001	24.34	3.85–153.92	0.001
	**Wealth quintile**	**Q1**	26.67 (17.47–38.46)	1			1		
		**Q2**	25.22 (14.84–39.48)	0.93	0.66–1.30	0.7	0.86	0.62–1.19	0.3
		**Q3**	24.23 (16.32–34.40)	0.88	0.62–1.25	0.5	0.9	0.61–1.32	0.6
		**Q4**	19.40 (13.56–26.99)	0.66	0.41–1.06	0.08	0.61	0.38–0.98	0.04
		**Q5**	12.55 (9.35–16.65)	0.39	0.23–0.67	0.001	0.41	0.24–0.69	0.002
	**PCR positive**		78.65 (60.30–89.94)	15.79	6.99–35.65	< .001	11.79	4.72–29.44	< .001
	**HH Head knows malaria can be prevented**		17.58 (12.65–23.89)	0.65	0.47–0.91	0.01	0.69	0.50–0.96	0.02
	**HH owns sufficient ITNs/LLINs**		32.35 (19.40–48.72)	2.28	1.21–4.31	0.01	2.28	1.16–4.48	0.02
**D3**	**Sex**	**Female**	9.15 (5.39–15.11)	1			1		
		**Male**	7.10 (4.60–10.81)	0.76	0.48–1.20	0.2	0.72	0.44–1.20	0.2
	**Age group**	**<5**	1.88 (0.25–12.83)	1			1		
		**5 to 14**	3.74 (1.87–7.34)	2.03	0.39–10.54	0.4	2.24	0.38–13.22	0.4
		**15+**	9.55 (6.02–14.81)	5.52	0.72–42.16	0.1	6.15	0.69–54.93	0.1
	**Quintile**	**Q1**	10.09 (5.08–19.05)	1			1		
		**Q2**	8.17 (4.65–13.96)	0.79	0.40–1.58	0.5	0.78	0.39–1.54	0.5
		**Q3**	10.90 (7.24–16.10)	1.09	0.58–2.05	0.8	0.98	0.51–1.88	1
		**Q4**	7.28 (4.18–12.37)	0.7	0.36–1.37	0.3	0.61	0.30–1.25	0.2
		**Q5**	3.68 (1.39–9.36)	0.34	0.13–0.87	0.03	0.27	0.12–0.63	0.004
	**Forest goer**		21.47 (11.67–36.14)	3.17	1.67–5.99	0.001	2.49	1.39–4.48	0.004
	**HH owns sufficient ITNs/LLINs**		20.92 (12.09–33.73)	3.25	1.63–6.49	0.002	3.5	1.94–6.33	< .001
	**HH Head heard BCC in previous 6 months**		13.33 (7.27–23.18)	1.83	1.00–3.36	0.05	1.91	1.14–3.22	0.02
**D4**	**Sex**	**Female**	28.48 (23.48–34.09)	1			1		
		**Male**	32.63 (26.39–39.56)	1.22	0.99–1.50	0.07	1.53	1.24–1.90	< .001
	**Age group**	**<5**	1.20 (0.26–5.26)	1			1		
		**5 to 14**	6.70 (5.17–8.65)	5.93	1.23–28.65	0.03	4.5	0.86–23.47	0.07
		**15+**	41.34 (33.86–49.24)	58.17	11.35–298.10	< .001	45.76	8.28–252.77	< .001
	**Area**	**Rural**	31.06 (25.37–37.38)	2.04	1.48–2.80	< .001	1.91	1.18–3.09	0.01
	**Quintile**	**Q1**	29.90 (22.79–38.13)	1			1		
		**Q2**	30.62 (23.97–38.18)	1.03	0.64–1.67	0.9	0.9	0.54–1.53	0.7
		**Q3**	34.03 (23.43–46.52)	1.21	0.67–2.18	0.5	1.02	0.55–1.86	1
		**Q4**	27.98 (20.82–36.47)	0.91	0.53–1.55	0.7	0.54	0.30–0.99	0.05
		**Q5**	18.36 (11.83–27.38)	0.53	0.28–1.01	0.05	0.44	0.21–0.94	0.03
	**Slept under LLIN previous night**		34.21 (28.34–40.61)	1.43	1.06–1.92	0.02	1.63	1.17–2.27	0.005

**Table 5:** Crude and adjusted odds ratios for *P*. *vivax* seropositivity per domain.

				Crude OR			Adjusted OR		
			Prevalence % (95% CI)	OR	95% CI	p-value	OR	95% CI	p-value
**D1**	**Sex**	**Female**	14.03 (10.32–18.78)	1			1		
		**Male**	14.88 (11.77–18.65)	1.07	0.91–1.26	0.4	1.13	0.95–1.34	0.2
	**Age group**	**<5**	3.07 (1.06–8.56)	1			1		
		**5 to 14**	4.33 (2.00–9.12)	1.43	0.37–5.53	0.6	2.13	0.61–7.45	0.2
		**15+**	17.67 (13.18–23.26)	6.76	2.20–20.81	0.001	9.89	3.27–29.94	< .001
	**Wealth quintile**	**Q1**	17.17 (11.81–2429)	1			1		
		**Q2**	15.30 (10.77–21.28)	0.87	0.61–1.25	0.4	0.88	0.60–1.31	0.5
		**Q3**	15.81 (11.24–21.76)	0.91	0.56–1.46	0.7	0.9	0.51–1.59	0.7
		**Q4**	12.23 (8.42–17.43)	0.67	0.39–1.16	0.1	0.64	0.34–1.22	0.2
		**Q5**	9.96 (7.08–13.84)	0.53	0.31–0.92	0.02	0.51	0.27–0.97	0.04
	**PCR positive**		37.23 (27.25–48.44)	3.73	2.18–6.39	< .001	3.11	1.86–5.20	< .001
	**Attended school**		12.96 (9.77–17.00)	0.61	0.40–0.93	0.02	0.60	0.38–0.94	0.03
**D2**	**Sex**	**Female**	7.68 (5.56–10.52)	1			1		
		**Male**	9.85 (6.58–14.50)	1.31	1.00–1.72	0.05	1.44	1.09–1.92	0.01
	**Age group**	**<5**	3.65 (1.36–9.41)	1			1		
		**5 to 14**	4.33 (2.35–7.84)	1.19	0.53–2.67	0.7	2.09	0.94–4.64	0.07
		**15+**	9.88 (6.89–13.96)	2.89	0.99–8.45	0.05	5.22	1.59–17.15	0.008
	**PCR positive**		48.67 (37.06–60.43)	10.95	5.90–20.31	< .001	9.43	4.37–20.33	< .001
	**Attended school**		7.59 (5.29–10.78)	0.53	0.36–0.80	0.003	0.59	0.38–0.92	0.02
	**Slept under LLIN previous night**		13.08 (9.56–17.64)	1.88	1.23–2.88	0.005	1.94	1.24–3.06	0.005
	**HH head knows sleeping under net is prevention method**		6.59 (4.70–9.15)	0.58	0.45–0.76	< .001	0.64	0.47–0.86	0.004
**D3**	**Sex**	**Female**	2.72 (1.22–5.95)	1			1		
		**Male**	3.79 (1.57–8.85)	1.41	0.99–2.02	0.06	1.45	1.02–2.05	0.04
	**Age (per year)**		n/a	1.02	1.01–1.03	0.004	1.02	1.01–1.03	0.002
	**Current fever**		11.24 (2.62–37.36)	4.06	1.36–12.17	0.01	4.11	1.50–11.26	0.008
	**HH Head attended school**		2.88 (1.21–6.71)	0.55	0.35–0.87	0.01	0.62	0.40–0.96	0.03
**D4**	**Sex**	**Female**	18.15 (14.17–22.94)	1			1		
		**Male**	20.55 (15.62–26.55)	1.17	1.01–1.35	0.04	1.14	0.97–1.36	0.1
	**Age group**	**<5**	2.24 (0.47–9.96)	1			1		
		**5 to 14**	7.15 (3.92–12.69)	3.36	0.74–15.26	0.1	2.19	0.60–9.18	0.3
		**15+**	25.06 (18.79–32.58)	14.56	2.74–77.41	0.003	9.82	1.79–54.00	0.01
	**Area**	**Rural**	20.04 (15.64–25.30)	3.28	1.89–5.70	< .001	2.51	1.56–4.05	0.001
	**PCR positive**		44.22 (34.09–54.85)	3.89	2.49–6.09	< .001	3.65	2.30–5.78	< .001
	**HH owns at least one ITN**		21.53 (16.69–27.32)	1.97	1.29–3.01	0.003	1.72	1.04–2.83	0.04
	**HH head knows sleeping under net is a prevention method**		24.68 (17.91–32.98)	1.57	1.16–2.11	0.004	1.42	1.15–1.74	0.002

### Association between incidence and prevalence

**Fig 6:**
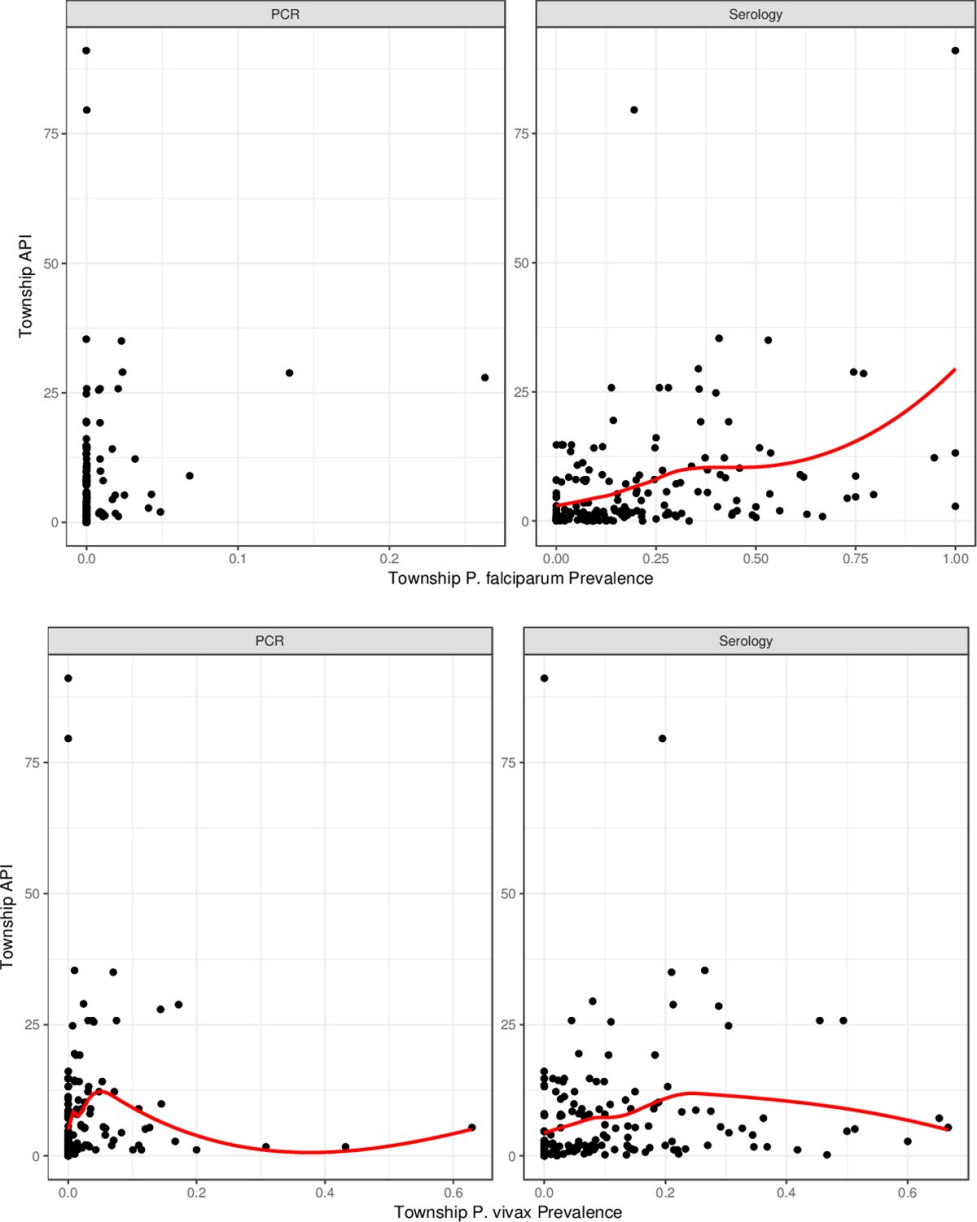
Association of prevalence and seroprevalence with incidence data, aggregated by township and split by *P*. *falciparum* (top) and *P*. *vivax* (bottom).

Township API data acquired during sampling was plotted against prevalence and seroprevalence figures aggregated by township (Fig 6). No clear trends were observed for either *P*. *vivax* and *P*. *falciparum* PCR prevalence and township API. A positive relationship was observed between increasing API and increasing seroprevalence to *P*. *falciparum*. However, the association between API and *P*. *vivax* was less clear with a positive association observed only until seroprevalence estimates of 25%.

## Discussion

The results of the first ever MIS in Myanmar reveal programmatically important epidemiological findings not apparent in routine surveillance data. Use of RDTs, now a common tool for identifying malaria infection in the field, only detected a tiny fraction of infections identified by PCR. RDTs can be useful for diagnosis of clinical disease but, as demonstrated here, are not a good surveillance tool, particularly in a context where asymptomatic and sub-patent infection may be highly prevalent [22]. Use of PCR, however, was able to detect a low-level national prevalence of just 0.74% for all-species Plasmodium infection, or 0.17% and 0.52% for species-specific *P*. *falciparum* and *P*. *vivax* infection, respectively. However, these overall estimates mask huge variation between the domains which ranged from an all-species prevalence of 10.96% in Domain 4 to 0.19% in Domain 3. Species-specific prevalence figures show that *P*. *falciparum* infections were relatively low and uniform across the country whereas *P*. *vivax*, made up the vast majority of infection in all domains and accounted for the observed heterogeneity. Given that serology captures both current and past infection, seroprevalence was expectedly higher at 16.01%, but showed similar variation between domains. In contrast to PCR data, the dominant Plasmodium species detected by serology was *P*. *falciparum*, including in young children. In comparing API data with prevalence and seroprevalence, where API was <25 per 1000 population, serology was able to capture a wider range of API variability than PCR, particularly for *P*. *falciparum* which showed a strong positive correlation between API with seroprevalence, although there was significant variation. Although there was an initial upward trend in API with *P*. *vivax* seroprevalence, this correlation was no longer apparent at prevalences greater than 25%.

The dominance of *P*. *vivax* prevalence is in contrast to routine surveillance data, which detects incident symptomatic clinical cases only and places *P*. *falciparum* as the major malaria species (75%) in Myanmar [11]. This is to be expected since *P*. *falciparum* is associated with higher parasite densities and more serious clinical outcomes than *P*. *vivax*, and as such leads to the poor correlation of *P*. *vivax* prevalence with API data we have shown [23, 24]. Whether this reservoir is important for transmission is under debate. While some studies have shown that asymptomatic and submicroscopic infections contribute to transmission, others have shown very limited infectivity to mosquitoes, particularly in the Asian transmission context [25–27]. A recent study, however, has suggested mosquito infection rates show positive correlation with blood parasitaemia when *P*. *vivax* parasitaemia is >10 parasites/ul, and that this may be the optimal diagnostic threshold since mosquito infection is rare at levels below this threshold [28]. Moreover, *P*.*vivax* infections tend to reach peak gametocytaemia prior to symptoms and are infectious before treatment this coupled with the relapse of infection due to their dormant liver stage hypnozoites, means transmissibility is difficult to predict [29]. Programmatically this is important because to achieve malaria elimination these relapses of infection will need to be targeted to remove the *P*. *vivax* infection reservoir. In practice this will be a challenge given that sub-clinical infections are poorly detected by RDTs and the only currently available radical cure for *P*. *vivax*, Primaquine, can be fatal in individuals with G6PD deficiency [30, 31]. *P*. *vivax* also doesn’t respond as well to the mainstays of vector control—ITNs/LLINs and IRS—largely due to its hypnozoite stage and association with outdoor and early biting vectors [32, 33]. Personal protection tools against outdoor biting mosquitoes are required that are effective and affordable for the target populations.

The higher seropositivity from *P*. *falciparum* may be due to the timing of data collection, much of which was after the transmission season, or because the ELISA assay used was originally developed to detect responses to *P*. *falciparum* antigens and thus may be more sensitive in detecting those. *P*. *falciparum* also circulates at higher parasite densities that induce higher antibody levels than *P*. *vivax* [34] and this is likely to induce more detectable antibody levels. Further analyses could be conducted to run the assay at higher serum concentrations against more antigenic targets to optimize *P*. *vivax* antibody detection [35].

The difference exhibited in prevalence between domains (API strata) supports the stratification design of the survey and is important to consider in the design of future surveys to capture important heterogeneities between regions. It also highlights the need for the Myanmar NMCP to be able to stratify its surveillance and approaches to interventions to suit the different transmission intensities in the country, including in the types of risk factors used to target high-risk population groups [36]. Moreover, the variation in prevalence was high even between individual clusters, supporting the need for village level stratification of malaria risk and village level case surveillance, particularly to identify hotspots and areas of drug resistance for a rapid intervention response. Domain 3, represents a zone approaching elimination in which new strategies for elimination could be implemented for the rest of the country to follow. Interestingly, across all domains, higher odds of malaria infection were associated with greater household net ownership and/or use, as well as knowledge of malaria and prevention methods. This seems counterintuitive and is likely because net distribution and behaviour change activities are targeted to areas of Myanmar with higher malaria caseloads and transmission.

Despite their differences, the use of serology in combination with PCR was firstly able to enrich findings by mapping areas (village data aggregated geographically) of potential high transmission that would have been missed by looking at infection prevalence results alone. In addition by comparing areas of high seropositivity (suggesting areas of past transmission) with areas of PCR positivity (current transmission) it is also possible to explore areas of potential high receptivity to complement surveillance efforts and guide programmes on optimal implementation of malaria control interventions. These higher transmission areas appear to be largely concentrated in north-west Myanmar, in the region of Sagaing, south-east in Rakhine and in the south-western tip of Myanmar in Mon, Kayin and Thanintharyi. Interpreting these areas as sites of high transmission requires the assumption that the infections were transmitted in the same location as that in which the diagnosis took place, which may not always be the case, particularly in areas with high population mobility. Although not presented here, basic travel history information was collected as part of the MIS questionnaire and only seven individuals had travelled outside the same state/region as their household in the previous six months. All other identified travellers had stayed away from home overnight but remained within the same state/region. Furthermore, whilst seroprevalence in children aged less than five years for both *P*. *falciparum* (2.33%) and *P*. *vivax* (1.41%) was low some had notable antibody responses, which, since young children are less likely to have travelled, indicates possible local and recent transmission (Fig 3). Future work might include antibody responses to recently described antigens that allow more discriminatory assessments of time since infection and sero-incidence [37, 38].

Secondly, serology provided an increased sample size of infections with which to analyse risk factors, improving uncertainty around OR estimates. This is useful programmatically to guide best targeting of resources, particularly as resources become more finite following reduction in caseloads. The recent National Strategic Plan has rightly specified activities for these risk groups such as, introducing quarterly malaria screening and additional LLINs for pregnant women through antenatal care services; emphasising case detection and treatment for forest-goers and mobile groups and engaging NGO networks in distribution of ‘forest-packages’ for forest-goers; and monitoring arrival of mobile population groups into communities by VHVs [3].

The findings offer important insights into malaria epidemiology in Myanmar, though the survey does have certain limitations. The proportion of non-response from household members was higher than anticipated and disproportionately consisted of males, travellers and children aged less than five years across all domains with the exception of D4 that had a younger population structure than the other three domains. Males and travellers were identified as risk groups and thus overall prevalence and seroprevalence may be slightly underestimated. Although attempts to revisit households were made, missing a certain proportion of household members is unavoidable and is expected to miss more mobile population groups. In contrast, pregnant women were overrepresented since, assumedly, they are more likely to be at home during day time. Despite not reaching the desired sample size, the loss in power to calculate the prevalence estimates was negligible. Finally, comparison of API and prevalence data is limited because API was only available at township level and did not include infections that might have been treated outside the formal health system. For comparison, we aggregated prevalence to township but townships are thus only represented by one or two villages, which with high amount of transmission heterogeneity may not be accurate. Work is ongoing in the country to measure and report API at village level; once this is achieved better understanding of the association between these two measures could be elucidated.

Despite these limitations, the survey presents the first nationwide malaria prevalence baseline findings for the Myanmar NMCP and partners as they move further toward malaria pre-elimination and elimination goals. The utility of these findings would be maximised if future surveys were to be regularly conducted from which to measure progress toward elimination of malaria. Use of PCR and particularly serology would enable monitoring of changes in transmission intensities to inform targeting of future interventions and ultimately certify elimination status [39]. Identified hotspots should be further investigated to determine reasons for high prevalence and where exactly transmission is occurring, with appropriate interventions put in place with urgency.

## Supporting information

S1 Table Number and percentage of household members (20,638) that provided blood samples for PCR and serological analysis by demographic factor. (DOCX)
