# Supplementary material for: Prevalence and seroprevalence of Plasmodium infection in Myanmar reveals highly heterogeneous transmission and a large hidden reservoir of infection
Source: PLoS One. 2021 Jun 9;16(6):e0252957. doi: 10.1371/journal.pone.0252957 (PMC8189444; doi:10.1371/journal.pone.0252957)
Supplement: S1 Table — (DOCX) [file pone.0252957.s001.docx]

S1 Table: Number and percentage of household members within each category that provided blood samples for PCR and serological analysis by demographic factor. Total number of household members (n=20,638).

|  | **HH members with PCR sample (n = 13,716)** | | **HH members with serology sample (n = 11,653)** | |
| --- | --- | --- | --- | --- |
|  | **n** | **% that provided samples in that category (95%CI)** | **n** | **% that provided samples in that category (95%CI)** |
| **Domain** |  |  |  |  |
| 1 | 5685 | 66.9 (62.9-70.7) | 4685 | 55.31 (50.7-59.9) |
| 2 | 3120 | 68.4 (64.2-72.4) | 2724 | 60.13 (55.6-64.5) |
| 3 | 2254 | 69.2 (63.1-74.7) | 2020 | 61.70 (55.5-67.5) |
| 4 | 2657 | 63.2 (54.2-71.3) | 2224 | 52.63 (42.0-63.0) |
| **Quintile** |  |  |  |  |
| 1 | 3803 | 68.2 (62.6-73.2) | 3264 | 62.33 (57.5-67.0) |
| 2 | 2229 | 68.9 (61.1-75.8) | 1906 | 60.74 (54.4-66.7) |
| 3 | 2530 | 70.7 (65.3-75.6) | 2103 | 61.71 (56.2-66.9) |
| 4 | 2715 | 69.2 (63.7-74.2) | 2270 | 59.69 (52.7-66.3) |
| 5 | 2439 | 67.2 (61.9-72.1) | 2110 | 59.99 (53.8-65.9) |
| **Area** |  |  |  |  |
| Urban | 2257 | 62.7 (54.5-70.2) | 1907 | 53.98 (44.8-62.9) |
| Rural | 11227 | 70.1 (65.8-74.1) | 9531 | 62.27 (57.8-66.5) |
| **Sex** |  |  |  |  |
| Male | 5964 | 63.8 (59.4-68.0) | 5090 | 56.57 (52.3-60.7) |
| Female | 7723 | 73.5 (69.5-77.2) | 6542 | 64.87 (60.5-69.0) |
| **Age group** |  |  |  |  |
| <5 | 757 | 51.1 (42.0-60.0) | 463 | 30.98 (24.3-38.6) |
| 5 to 14 | 2886 | 65.4 (56.3-73.5) | 2323 | 55.51 (47.0-63.7) |
| 15+ | 10034 | 72.5 (69.4-75.4) | 8846 | 66.09 (62.4-69.6) |
| **Fever previous 2 weeks** |  |  |  |  |
| Yes | 663 | 77.6 (70.3-83.6) | 542 | 66.56 (59.5-73.0) |
| No | 13053 | 68.3 (64.4-72.0) | 11111 | 60.51 (56.5-64.4) |
| **Forest-goer** |  |  |  |  |
| Yes | 499 | 57.9 (49.3-66.1) | 437 | 55.18 (46.6-63.5) |
| No | 13217 | 69.2 (65.1-73.0) | 11216 | 61.01 (56.8-65.1) |
| **Visitor** |  |  |  |  |
| Yes | 150 | 58.4 (42.7-72.6) | 117 | 46.33 (33.2-60.0) |
| No | 13566 | 68.9 (64.9-72.7) | 11536 | 61.00 (56.9-65.0) |
| **Pregnant woman** |  |  |  |  |
| Yes | 210 | 94.1 (86.0-97.6) | 183 | 91.27 (83.7-95.5) |
| No | 13504 | 68.6 (64.6-72.3) | 11468 | 60.57 (56.5-64.5) |
| **Traveller** |  |  |  |  |
| Yes | 1370 | 50.4 (45.8-55.0) | 1233 | 46.58 (41.8-51.4) |
| No | 12346 | 72.5 (68.4-76.3) | 10420 | 63.72 (59.5-67.7) |
| **Attended school** |  |  |  |  |
| Yes | 10445 | 70.5 (66.4-74.2) | 9052 | 63.40 (59.0-67.6) |
| No | 3256 | 61.5 (56.2-66.6) | 2591 | 49.09 (44.6-53.7) |
| **HH head attended school** |  |  |  |  |
| Yes | 9457 | 68.6 (64.3-72.5) | 8130 | 61.12 (56.7-65.4) |
| No | 4000 | 69.8 (65.2-74.1) | 3325 | 60.00 (55.5-64.3) |
